# A realistic arteriovenous dialysis graft model for hemodynamic simulations

**DOI:** 10.1371/journal.pone.0269825

**Published:** 2022-07-21

**Authors:** Sjeng Quicken, Barend Mees, Niek Zonnebeld, Jan Tordoir, Wouter Huberts, Tammo Delhaas

**Affiliations:** 1 Department of Biomedical Engineering, Cardiovascular Research Institute Maastricht, Maastricht, the Netherlands; 2 Department of Biomedical Engineering, Eindhoven University of Technology, Eindhoven, the Netherlands; 3 Department of Vascular Surgery, Maastricht University Medical Centre, Maastricht, the Netherlands; Universidade de Lisboa Instituto Superior Tecnico, PORTUGAL

## Abstract

**Objective:**

The hemodynamic benefit of novel arteriovenous graft (AVG) designs is typically assessed using computational models that assume highly idealized graft configurations and/or simplified boundary conditions representing the peripheral vasculature. The objective of this study is to evaluate whether idealized AVG models are suitable for hemodynamic evaluation of new graft designs, or whether more realistic models are required.

**Methods:**

An idealized and a realistic, clinical imaging based, parametrized AVG geometry were created. Furthermore, two physiological boundary condition models were developed to represent the peripheral vasculature. We assessed how graft geometry (idealized or realistic) and applied boundary condition models of the peripheral vasculature (physiological or distal zero-flow) impacted hemodynamic metrics related to AVG dysfunction.

**Results:**

Anastomotic regions exposed to high WSS (>7, ≤40 Pa), very high WSS (>40 Pa) and highly oscillatory WSS were larger in the simulations using the realistic AVG geometry. The magnitude of velocity perturbations in the venous segment was up to 1.7 times larger in the realistic AVG geometry compared to the idealized one. When applying a (non-physiological zero-flow) boundary condition that neglected blood flow to and from the peripheral vasculature, we observed large regions exposed to highly oscillatory WSS. These regions could not be observed when using either of the newly developed distal boundary condition models.

**Conclusion:**

Hemodynamic metrics related to AVG dysfunction are highly dependent on the geometry and the distal boundary condition model used. Consequently, the hemodynamic benefit of a novel graft design can be misrepresented when using idealized AVG modelling setups.

## 1 Introduction

Despite multiple interventions to preserve function, AVGs for hemodialysis typically lose patency within two years due to neointimal hyperplasia (NIH) near the graft-vein anastomosis [1, 2]. NIH causes stenosis, low flow and ultimately thrombosis and patency loss. Disturbed blood flow and resulting non-physiological (oscillatory) wall shear stress (WSS) at the graft-vein anastomosis are believed to be the main triggers for NIH development [1, 2].

Hemodynamically optimized graft designs have been proposed to increase AVG longevity by reducing hemodynamic disturbances near the graft-vein anastomosis [3]. The hemodynamic benefit of these grafts is typically assessed and demonstrated using computational fluid dynamics (CFD) simulations [4–11], because, as opposed to experimental *in vitro* techniques, CFD modelling allows for straightforward, high resolution assessment of WSS parameters and for simultaneous evaluation of multiple graft designs. Despite promising results in CFD studies, *in vivo* patency rates of AVGs have not dramatically increased and clinical adaptation of novel graft designs is limited [3, 12].

A possible cause for the discrepancy between the CFD based expectations and clinical reality is that the applied CFD strategies often use simplified AVG geometries and/or boundary conditions. AVG geometries are often highly idealized and/or only take into account the graft-vein anastomosis [13]. An advantage of these idealized geometries is that they can be easily parametrized to allow for straightforward implementation of new graft designs, something that is more difficult to do in patient-specific geometries. Furthermore, though often great care is taken to prescribe realistic flow patterns through the main in- and outlet of the geometry [4–9], flow to and from the distal vasculature (*i*.*e*. the hand) is often regarded negligible compared to the graft flow and set to zero [4, 5, 8, 9]. While such boundary conditions might have little influence on bulk flow and WSS magnitude, it might greatly affect derived WSS metrics such as the oscillatory shear index (OSI) [5]. Though the use of idealized AVG geometries and zero-flow boundary conditions may be tempting due to lack of clinical data to inform the model or because the study design requires highly modifiable AVG representations, it remains that hemodynamics are highly susceptible to the applied boundary conditions [14] and to geometric vessel characteristics [15–18]. As such, the applicability of these simplified approaches for evaluating hemodynamic graft performance might be limited.

The aim of this study was to assess the importance of using realistic geometries and boundary conditions when evaluating AVG hemodynamics. For this purpose, blood flow characteristics related to graft dysfunction were compared between AVG models using either realistic or highly idealized geometries and/or boundary conditions.

## 2 Materials and methods

Patient data used in this study were obtained from clinical follow-up for monitoring graft function and were measured at the Maastricht University Medical Centre (*Maastricht*, *the* Netherlands). A waiver for ethical approval for this study was obtained from the local medical ethical committee.

### 2 1 AVG geometries

#### 2.1.1 Realistic AVG geometry creation framework

**Fig 1:**
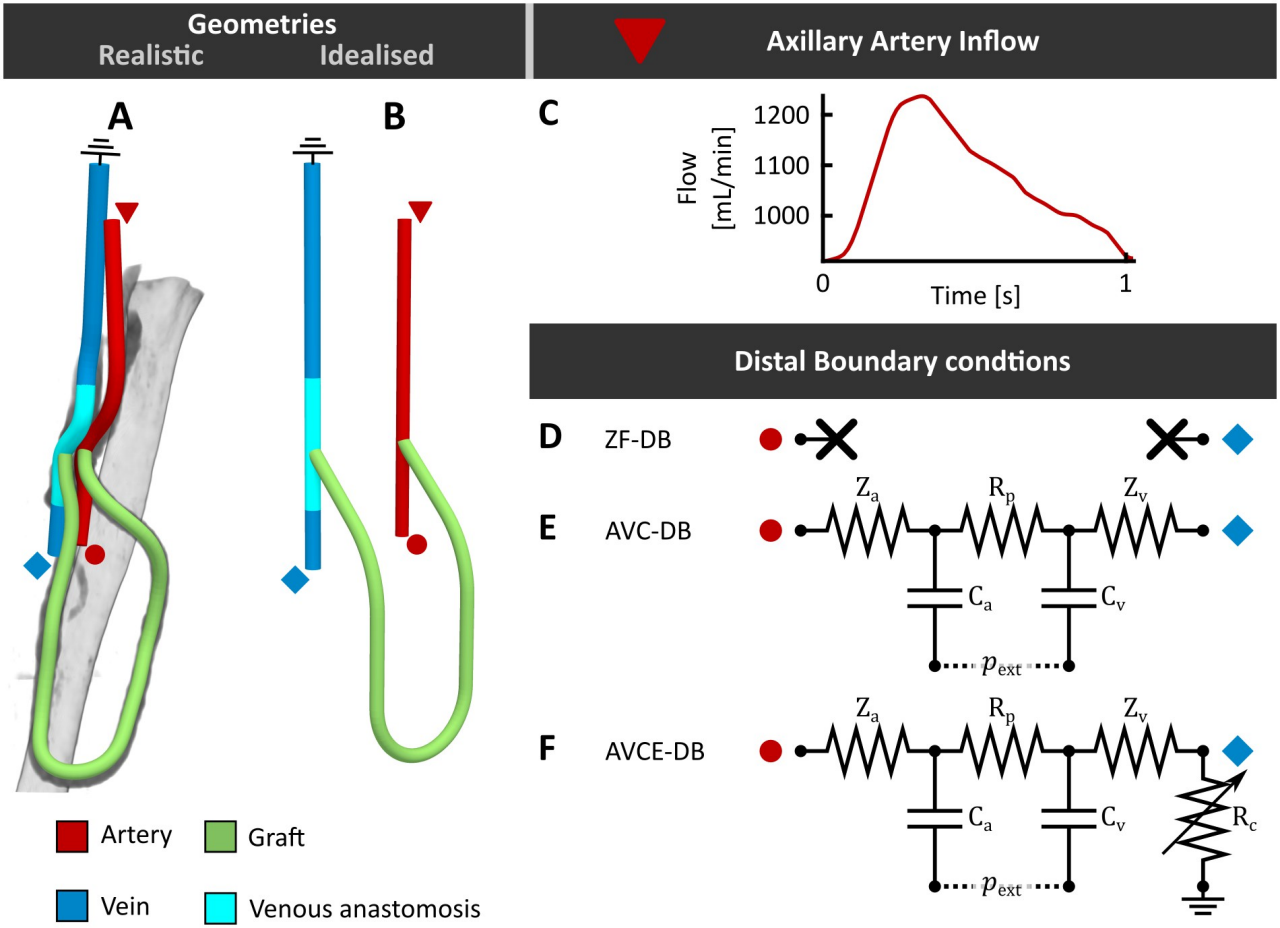
An overview of the realistic (A) and idealized (B) AVG model. Flow prescribed at the arterial inlet is presented in subfigure C. The distal boundary condition models that could be prescribed are presented in subfigures D—F. Note that the colored symbols in subfigures C—F correspond to the symbols near the boundaries in A & B and indicate where each boundary condition was prescribed.

A realistic, parametrized AVG geometry was created from a 15 months-postoperative computed tomography angiography (CTA) scan (performed for diagnostic purposes) of a single patient with an axillary-artery to axillary-vein upper-arm loop AVG (Fig 1A). For medical-ethical reasons only diagnostic imaging studies as standard of care were available for this research. Clinical evaluation of the CTA revealed a non-significant stenosis near the venous anastomosis. Because the stenosis was not considered clinically relevant, it was assumed that it had not caused any major remodeling of the vessel prior to imaging that may have resulted in a shift of the vessel path with respect to its original configuration.

The graft and adjacent blood vessels were segmented from the CTA data using the software package VMTK [19]. Graft configuration and vessel shape were subsequently defined by computing the vessel’s centerlines, which were imported into SolidWorks 2018 (*Dassault Systèmes*, *Vélizy-Villacoublay*, *France*). Vessels with circular cross-sections were imposed onto the centerlines to define the inner lumen of the artery, vein and graft. Arterial and venous diameters were measured according to standardized techniques (inner wall diameter, transverse ultrasound probe position) on a two weeks-preoperative ultrasound examination for AVG surgery planning and set to 6.6 mm and 7.7 mm, respectively. Graft diameter was chosen to be 6 mm. The length of the venous and arterial segments proximal to the anastomosis were cut to 7.5 times the respective diameter, whereas distal to the anastomosis the venous and arterial segments were cut to 3.25 times the respective diameter. Straight flow extensions were subsequently added to double the length of the distal and proximal arterial and venous vessel segments, in order to reduce the influence of possible boundary artefacts on the simulation results [20].

#### 2.1.2 Idealized AVG model

An idealized AVG geometry was created in SolidWorks 2018 (Fig 1B). Arterial and venous segments were modelled as straight tubes. The graft anastomosis was defined as fully in-plane with the autologous vessels and followed a path that could be defined in three orthogonal planes. Arterial and venous anastomotic angles were set to 45° [4, 7, 9, 10]. All vessel lengths and diameters, including those of the graft, were set equal to those prescribed in the realistic AVG geometry.

### 2.2 Boundary conditions

#### 2.2.1 Arterial inlet and venous outlet

A pulsatile velocity profile was prescribed at the arterial inlet (Fig 1C). It was assumed that, to maintain sufficient perfusion of the peripheral vasculature, time averaged blood flow towards the hand would not be influenced by AVG creation. Consequently, the flow at the arterial inlet was assumed to be equal to the sum of the 7 week-postoperative pulsatile graft flow (average flow: 990 mL/min) and the 2 week-preoperatively measured average brachial artery flow (*i*.*e*. 73 mL/min), resulting in an averaged flow of 1.06×10^3^ mL/min. Both preoperative and postoperative flow were assessed using clinical Doppler ultrasound measurements. Since blood flow characteristics in our modelling setup depend on the pressure drop over the AVG and not on absolute prescribed pressure values, pressure at the venous outlet was assumed constant and was set to 0 mmHg.

#### 2.2.2 Distal boundary conditions

Three different sets of boundary conditions with different levels of complexity were applied to the distal model boundaries of the AVG models.

The simplest distal boundary condition model assumed that the flow to and from the peripheral vasculature was negligible compared to the graft flow. Consequently, a zero-flow distal boundary condition model (ZF-DB) was prescribed at the arterial outlet and the venous inlet (Fig 1D). This boundary condition corresponds to the simplified approach often used for AVG simulations [4, 5, 8, 9].

A second boundary condition model was developed to couple the arterial outlet to the venous inlet with a lumped-parameter model of the peripheral vasculature. In this arteriovenous coupling model (AVC-DB, Fig 1E), the distal arteries and veins were represented as compliant systems (C_a_ and C_v_, respectively) that were connected by a purely resistive microvasculature (R_p_). The lumped parameter model was coupled to the arterial outlet and venous inlet using their characteristic impedances (Z_a_ and Z_v_, respectively).

Finally, an extension of the arteriovenous coupling model (AVCE-DB, Fig 1F) was developed that allowed for splitting the returning flow from the peripheral vasculature over the venous segment of the CFD simulation and other collateral veins that might be present in reality. Here, collateral veins were represented by an adjustable resistor (R_c_) in parallel to the 3D simulated venous segment. In this study the value of R_c_ was updated at each time step, such that the flow was equally distributed over the collateral and the 3D simulated veins.

To ensure that the flow towards the peripheral vasculature corresponded to the preoperatively measured value for both arteriovenous coupling models, an initial CFD simulation was performed for both the realistic and the idealized AVG geometry to fit the value of the total peripheral resistance (*i*.*e*. *R*_tot_ = *Z*_a_ + *R*_p_ + *Z*_v_). During fitting, all compliances of the boundary condition model were set to zero and the average value of the axillary-artery flow was prescribed at the arterial inlet. After fitting, impedances and resistances in the arteriovenous model were determined as: R_p_ = 0.9 *R*_tot_ and *Z*_a_ = *Z*_v_ = 0.05 *R*_tot_ (Fig 1E and 1F). Finally, arterial and venous compliance were chosen such that *R*_p_*C*_a_ = *R*_p_*C*_v_ = 0.5s [21].

Both arteriovenous coupling models were implemented using the methods described in Kroon et al. [22]. An extensive description of the numerical implementation of both models is presented in Appendix A in the Supplementary Information S1 Appendix.

### 2.3 CFD simulations

Blood flow through the AVG geometries was simulated by solving the incompressible Navier-Stokes equations using the open source CFD solver OASIS [23] implemented in the finite element package FEniCS [24].

Blood was modelled as a Newtonian fluid with a dynamic viscosity of 3.5 Pa⋅s [25] and a density of 1050 kg/m^3^ [26]. Computational meshes of the AVG geometries were made in ICEM 18 (*Ansys*, *Canonsburg*, *PA*, *USA*). Mesh density was increased in a spherical region with a radius of 2.5 cm around both anastomoses to increase local solver accuracy. For the realistic geometry, a grid independent solution was obtained at 3.1×10^6^ second-order tetrahedral Taylor-Hood elements. The idealized geometry was meshed using the same meshing size settings as the realistic geometry and consisted of 2.8×10^6^ tetrahedral elements. The solver was progressed in time using time steps of 0.1 ms. Three cardiac cycles were simulated, of which the last one was used for analysis.

#### 2.3.1 Hemodynamic metrics for disturbed flow and non-physiological WSS

Hemodynamic metrics were defined to quantify disturbed flow and disturbed WSS in the anastomotic region. Although multiple hypotheses regarding the definition of disturbed WSS exist [27], here it was assumed that WSS outside the physiological range (0.1–7 Pa [28]), WSS sufficiently high to cause irreversible endothelial damage (>40 Pa [29]) and highly oscillatory WSS [30] were detrimental to graft longevity. Disturbed flow and non-physiological WSS were assessed over the total venous segment and in the venous perianastomotic region of all simulations. The venous perianastomotic region was defined as the venous segment ranging from 2.5 cm distal to 3.0 cm proximal to the venous anastomosis (Fig 1A and 1B).

Exposure to non-physiologically low WSS (<0.1 Pa [28]) was assessed by computing the time-averaged WSS magnitude (TAWSS):

$$\text{TAWSS}=\frac{1}{\text{T}}\int_{0}^{\text{T}}\left|\left|\vec{\tau }\left(t,\vec{x}\right)\right|\right|dt,$$ (1)

where $\vec{\tau }\left(t,\vec{x}\right)$ represents the local WSS vector and T the duration of the cardiac cycle.

Exposure to non-physiologically high (>7 Pa [28], ≤40 Pa) and very high WSS (>40 Pa [29]), where assessed using the time maximum WSS (WSS_max_):

$$\text{WS}{\text{S}}_{\text{max}}=max\;\left\{\left\|\vec{\tau }\left(t,\vec{\text{x}}\right)\right\|:t=0..T\right\}.$$ (2)

Finally, exposure to oscillating WSS was assessed using the oscillatory wall shear stress index (OSI) [31]:

$$\text{OSI}=\frac{1}{2}\left(1-\frac{\left\|\int_{0}^{T}\vec{\tau }(t,\vec{x})dt\right\|}{\int_{0}^{T}\left\|\vec{\tau }(t,\vec{x})\right\|dt}\right).$$ (3)

The OSI ranges between 0 (unidirectional WSS) and 0.5 (purely oscillatory WSS). A threshold of OSI > 0.25 was used to identify regions exposed to highly oscillatory WSS.

To quantify disturbed flow, regions of transitional to turbulent flow where identified using the root-mean-square (RMS) magnitude of high frequency velocity perturbations in the AVG model. Here, Reynolds decomposition was used to decompose the local velocity magnitude $u\left(t,\vec{x}\right)$ into the average velocity trend $\overset{-}{u}\left(\vec{x}\right)$ and high frequency perturbations $\tilde{u}\left(t,\vec{x}\right)$. Subsequently, ${\tilde{u}}_{\text{RMS}}\left(\vec{x}\right)$ was computed as the RMS value of $\tilde{u}\left(t,\vec{x}\right)$ over the last cardiac cycle. The development of transitional to turbulent flow along the venous segment was assessed using ${\tilde{u}}_{\text{RMS,50}}$, *i*.*e*. the cross-sectional median value of ${\tilde{u}}_{\text{RMS}}\left(\vec{x}\right)$ perpendicular to the centerline.

### 2.4 Simulations and analysis

All six combinations of geometry (realistic or idealized) and distal boundary condition model (ZF-DB, AVC-DB, AVCE-DB) were evaluated using CFD. The impact of the distal boundary condition model and the geometry on the observed hemodynamics were studied by comparing the metrics for non-physiological WSS and the magnitude of high frequency velocity perturbations. Furthermore, the effect of the geometry and boundary conditions on flow distribution over each outflow boundary and the total pressure drop over the geometry was assessed.

## 3 Results

### 3.1 Disturbed flow

**Fig 2:**
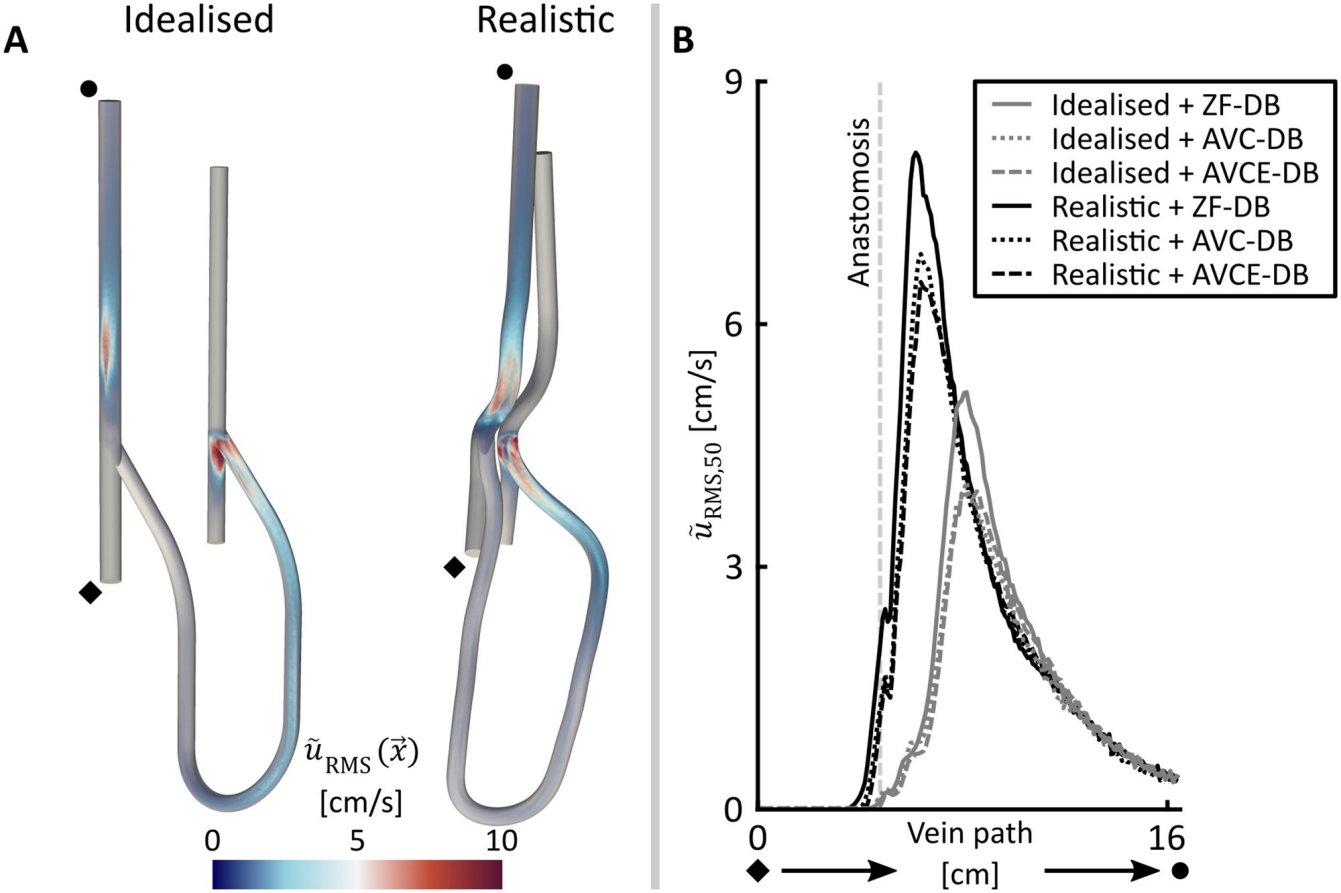
A: Overview of the magnitude of velocity perturbations in the idealized and realistic AVG geometries (both with distal boundary condition AVC-DB) B: Value of ${\tilde{u}}_{\text{RMS,50}}$ along the venous segment of both the idealized and the realistic AVG geometry.

We observed that the magnitude of high frequency flow perturbations (${\tilde{u}}_{\text{RMS}}\left(\vec{x}\right)$) was lower than 0.01 cm/sec in the proximal arterial segments of all simulations, indicating that flow was stable and laminar for the complete cardiac cycle (Fig 2A) This laminar flow was maintained up to 1 cm before the artery-graft anastomosis. Distal to the arterial anastomosis of the idealized geometry, flow showed stable 40–45 Hz oscillations for each distal boundary condition model as a result of vortex shedding. This behavior was not observed in any of the simulations employing the realistic AVG geometry. At the arterial inlet of the graft, ${\tilde{u}}_{\text{RMS}}\left(\vec{x}\right)$ increased up to 25 cm/sec, which gradually decreased along the path of the graft. At the venous anastomosis of all simulations a jet was observed that exited the graft and impinged on the venous floor. In the idealized AVG geometries, the jet broke down after approximately 2.5 cm proximal to the venous anastomosis, whereas in the realistic AVG geometry jet breakdown was observed at the curved segment proximal to the anastomosis. For all simulations jet breakdown coincided with an increase in the cross-sectional median value of ${\tilde{u}}_{\text{RMS}}\left(\vec{x}\right)\;({\tilde{u}}_{\text{RMS,50}}$) (Fig 2B). It was observed that, given the same distal boundary condition model, the maximum of ${\tilde{u}}_{\text{RMS,50}}$ was up to 1.7 times higher in the realistic geometry compared to the idealized one. Finally, the maximum value of ${\tilde{u}}_{\text{RMS,50}}$ was up to 13% higher for the simulations in which the peripheral flow was impeded (ZF-DB), compared to the simulations where AVC-DB or AVCE-DB were prescribed (Fig 2B).

### 3.2 Wall shear stress metrics

**Fig 3:**
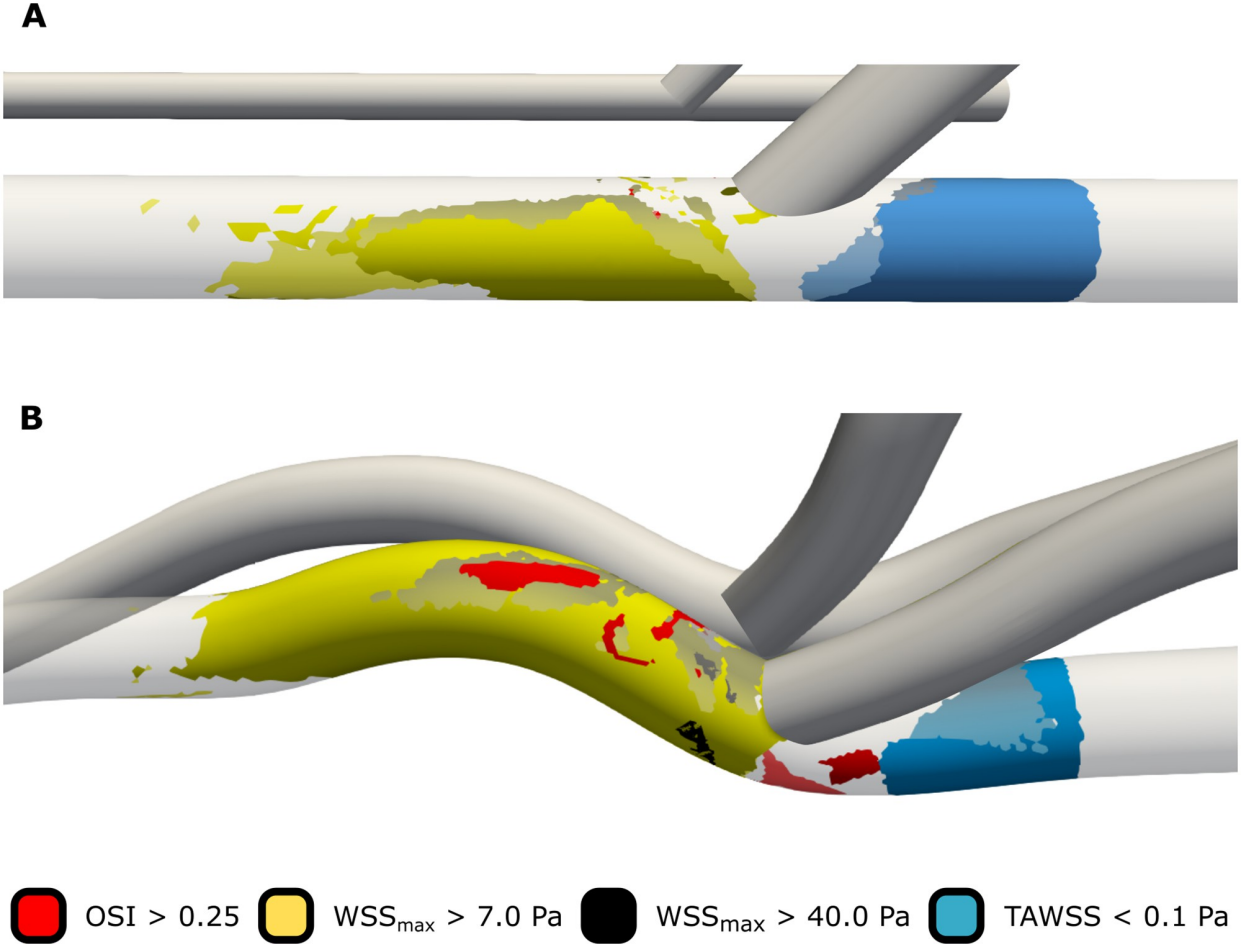
Comparison of the exposure to detrimental WSS characteristics in the venous anastomotic region between the idealized (A) and realistic (B) AVG simulations using the AVC-DB distal boundary condition model.

It was observed that AVG geometry had a large influence on the anastomotic and venous area exposed to low WSS (<0.1 Pa). Depending on the distal boundary condition model, the percentage of the anastomotic area exposed to low WSS was 20%–50% larger for the idealized AVG geometry than for the realistic one (Fig 3, Table 1). A similar trend was observed for the total venous segment exposed to low WSS, although differences between the geometries were smaller. Furthermore, it was observed that anastomotic region exposed to low WSS was up to 50% smaller in the simulations in which flow to the peripheral vasculature was impeded (ZF-DB), compared to those in which flow was regulated with an arteriovenous coupling model (AVC-DB and AVCE-DB). However, the total venous area exposed to low WSS did not change more than approximately 3% between different distal boundary conditions (Table 1).

**Table 1:** Overview of the metrics for disturbed hemodynamics in the anastomotic region and over the complete venous segment.

		Idealized			Realistic		
Distal boundary model		ZF-DB	AVC-DB	AVCE-DB	ZF-DB	AVC-DB	AVCE-DB
***Anastomotic region***							
*WSS*_*low*_	*[%]*	22.3	32.1	32.8	18.6	21.3	26.7
*WSS*_*high*_	*[%]*	39.3	33.5	35.9	56.7	51.5	54.3
*WSS*_*very high*_	*[%]*	0.0	0.0	0.0	1.9	1.1	1.1
*OSI*_*high*_	*[%]*	2.2	0.2	0.9	14.6	4.3	5.5
***Complete venous segment***							
*WSS*_*low*_	*[%]*	23.6	26.6	26.9	21.1	21.9	23.8
*WSS*_*high*_	*[%]*	15.4	11.8	13.0	21.8	20.9	21.2
*WSS*_*very high*_	*[%]*	0.0	0.0	0.0	0.6	0.4	0.4
*OSI*_*high*_	*[%]*	16.3	0.2	0.4	9.9	1.6	1.9

The area exposed to non-physiologically high WSS (>7 Pa) covered more than 50% of the total venous anastomosis of the realistic AVG geometry. In comparison, the anastomotic region exposed to high WSS was less than 40% in the idealized geometry (Fig 3, Table 1). Furthermore, a small region exposed to WSS in excess of 40 Pa was observed in the anastomotic region of the realistic AVG geometry simulations. Such a region did not exist in the CFD simulations of the idealized geometry.

Finally, the area exposed to highly oscillatory WSS (OSI > 0.25) made up maximally 2.2% of the venous anastomotic region of the idealized AVG geometry, whereas highly oscillatory WSS was much more present in the venous anastomotic region of the realistic AVG geometry (Fig 3, Table 1). When neglecting peripheral flow (ZF-DB), a considerable percentage of the idealized geometry’s total venous segment was exposed to highly oscillating WSS, which was mainly located at the distal venous segment. These regions disappeared when either of the distal arteriovenous coupling models (AVC-DB or AVCE-DB) were used (Table 1). Although less pronounced, similar observations were made for the realistic AVG geometry.

### 3.3 Flow distribution

**Fig 4:**
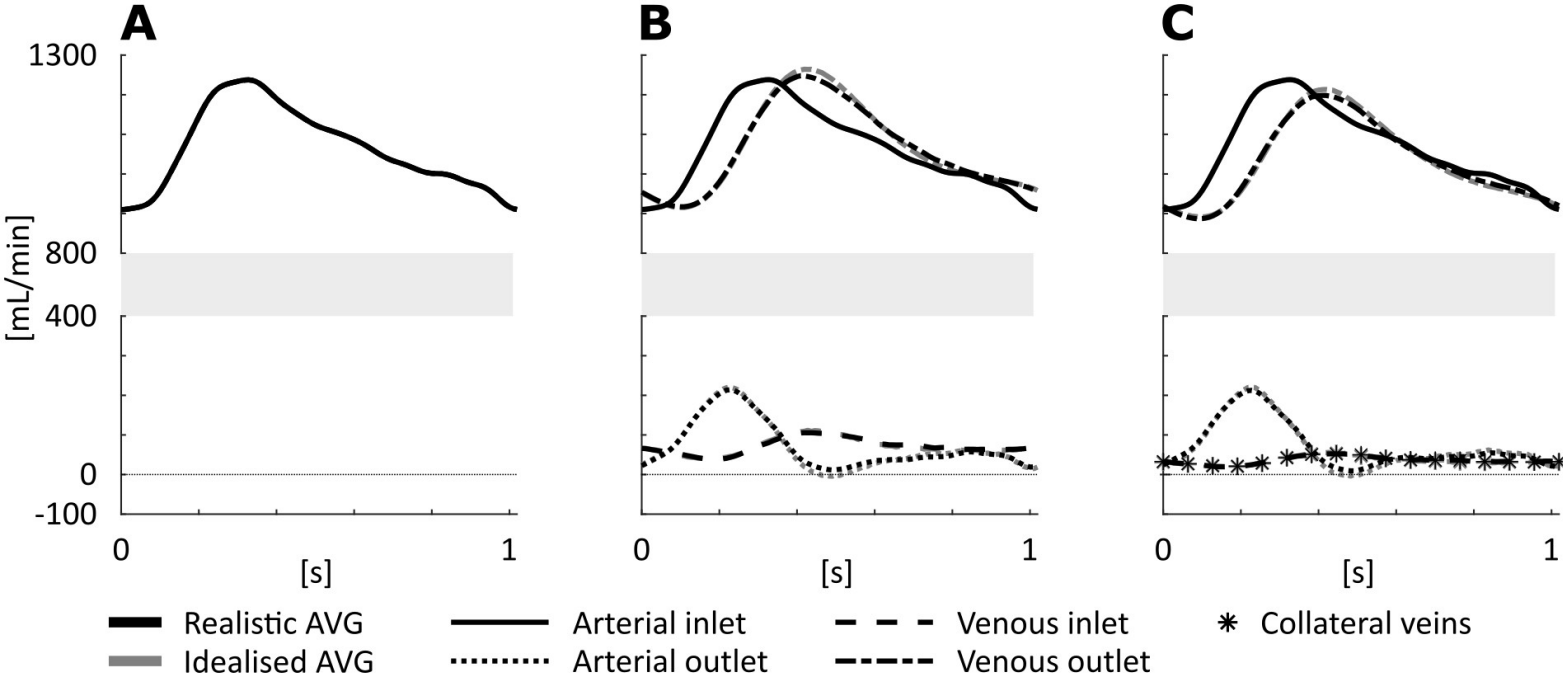
Overview of the flows over the in- and outlets of the 3D geometry and over the collateral veins for distal boundary condition model ZF-DB (A), AVC-DB (B) and AVCE-DB (C).

Differences in average flow distribution over the various in- and outlets of the idealized and realistic AVG geometries were within 1.5 mL/min of each other, when applying the same distal boundary conditions (Fig 4). For the simulations performed with ZF-DB, arterial inflow and venous outflow were equal in shape and magnitude (Fig 4A). When applying AVC-DB or AVCE-DB, a phase shift was observed between the arterial inlet and the venous outlet (Fig 4B and 4C). For both AVC-DB and AVCE-DB, flow at the arterial outlet showed an (almost) triphasic flow pattern, *i*.*e*. a fast acceleration during systole, followed by a slight retrograde or zero flow during early diastole, and slow antegrade flow for the remainder of the cardiac cycle. Flow through the venous inlet showed low pulsatility and was monophasic (Fig 4B and 4C). Average flow through the arterial outlet was 70–71 mL/min when applying either arteriovenous coupling model. For the simulations where the arteriovenous coupling model extended with collateral veins (AVCE-DB) was prescribed, time averaged flow was evenly distributed over the 3D simulated vein and the collateral veins (< 0.1 mL/min difference) (Fig 4C).

### 3.4 Pressure drop over the AVG geometry

Simulated pressure drops from the arterial inlet to the venous outlet were approximately 17%-18% higher within the realistic AVG geometry compared to those observed in the idealized one with the same distal boundary condition (12–13 mmHg vs 14–16 mmHg, respectively). Furthermore, when applying distal boundary condition ZF-DB, the pressure drop over the geometry was approximately 1 mmHg higher compared to boundary conditions AVC-DB and AVCE-DB.

It was observed that in the idealized geometry the pressure drop showed oscillations of approximately 45 Hz. This frequency coincided with the flow oscillations that where observed at the artery-graft anastomosis of the idealized AVG geometry.

## 4 Discussion

Novel graft designs that aim to improve hemodynamics at an AVG’s venous anastomosis are often evaluated with CFD simulations using highly idealized model set-ups. As a result of this idealized approach, observed hemodynamics might not be representative of reality, which might explain the limited clinical adaptation of novel graft designs [3, 12]. The aim of this study was to evaluate how using idealized AVG models can affect modelling results and to develop a modelling strategy that could serve as a physiological basis for evaluating novel grafts.

We demonstrated that complexity of hemodynamic metrics related to AVG dysfunction is considerably underestimated in idealized AVG geometries compared to the realistic ones. Since simplified models are often used in the development of new graft designs [4–9], the hemodynamic benefit of graft designs developed using these simplified models could be overestimated or generally misrepresented.

The methods proposed in this study allowed to create more realistic AVG parametrizations. As such, these parametrizations may help to evaluate new grafts under more realistic conditions. The technique proposed employs clinical data to extract vessel path and AVG configuration in order to define AVG parametrizations that more closely resemble the *in vivo* AVG configuration. Since vessel characteristics, such as diameter can be adjusted in the AVG parametrizations, new graft concepts can straightforwardly be implemented in the parametrizations. Subsequently, the new graft concepts can be evaluated in a model that more closely resembles the *in vivo* AVG configuration. An example of this approach has recently been presented in Quicken et al. [11], where the realistic AVG geometry used in this study was further extended with potentially relevant geometric features. A helical graft centerline and/or helical ridge in the lumen of the graft were added, parameterized by specification of a helical pitch and amplitude, and/or the width and height of the ridge. In addition, the axial rotation of the graft was considered as a parameter. By varying these geometric parameters multiple realistic AVG parametrizations can be created. Subsequently, an efficient optimization strategy was applied to identify the set of parameters that resulted in optimal values of predefined shear stress-derived metrics such as OSI, TAWSS.

Besides evaluating the hemodynamic impact of using realistic instead of idealized AVG geometries, also the impact of a realistic representation of the vasculature distal to the AVG was assessed. For this purpose, two new distal AVG boundary conditions were developed that mimicked the vasculature distal to the 3D modelled domain. Using these boundary conditions, the blood flow waveform at the arterial outflow was a triphasic, whereas venous flow was monophasic and showed considerably reduced pulsatility compared to the arterial flow. Since this behavior is expected in healthy individuals and because it has been demonstrated that in arteriovenous fistulas (AVF) flow distal to the anastomosis also retains its triphasic waveform [32], these results suggest that the proposed boundary condition models are physiologically accurate. Because the two newly developed boundary condition models resulted in similar anastomotic hemodynamic phenomena, the results from this study suggest that collateral veins may be ignored in future studies.

Higher velocity perturbation magnitudes and larger regions exposed to non-physiologically high WSS were observed when applying either of the arteriovenous coupling models instead of ZF-DB. Both the development of transitional to turbulent flow and the increase of local WSS magnitude are directly related to an increase in blood flow velocity. Consequently, this observation might be explained by the fact that local blood flow velocities in the observed venous anastomotic jet were higher when applying ZF-DB, because all flow was directed through the graft, instead of also to the peripheral vasculature.

A large region of highly oscillatory WSS was observed distal to the venous anastomosis when applying ZF-DB, which is probably a direct artefact from the applied boundary condition. These artefacts will likely also affect OSI derived metrics such as the relative residence time (RRT) [33]. Future studies on AVG hemodynamics should therefore preferably avoid using a boundary condition in which peripheral flow is impeded. If this is not possible due to the modelling setup, the region of high OSI should be acknowledged as a non-physiological artefact, resulting from the applied boundary condition.

### 4.1 Limitations and future research

Hemodynamics are considered to play a large role in AVG dysfunction. Nonetheless, the relation between hemodynamics and AVG dysfunction has not yet fully been elucidated [27]. In this study, WSS outside the physiological range and WSS sufficiently high to cause immediate endothelial damage, as well as highly oscillatory WSS and high frequency flow perturbations were considered to be detrimental to graft dysfunction. However, other metrics including, but not limited to, high temporal or spatial WSS gradients have also been linked to AVG dysfunction [27]. In addition, other shear-derived metrics such as transWSS [34] that have been demonstrated to be relevant for atherosclerosis, might also be relevant for NIH development. Hence, such metric could also be considered in future research on AVG dysfunction. Furthermore, other metrics for disturbed flow have been proposed that, compared to the high frequency flow perturbations-based approach used in this study, would allow for a more detailed analysis of the direct effect of disturbed flow on the vessel wall [35]. However, given the large impact of AVG parametrization and boundary conditions on the hemodynamic evaluated in this study, we hypothesize that also other hemodynamics metrics linked to AVG dysfunction will be affected by the AVG parametrization and boundary conditions used. If future research establishes strong evidence that hemodynamic metrics other than the ones evaluated in this study are linked to AVG dysfunction, more research should establish how modelling assumptions affect these parameters.

The imaging data used in this study was sourced from diagnostic investigations (duplex and CTA) performed as standard of care during preoperative work-up and follow-up of the AVG. As such, there is a date discrepancy between the used duplex and CTA data. This is off course not ideal but because postoperative flow conditions have stabilized 7 weeks postoperatively and in light of the absence of stenosis at both timepoints and the prosthetic nature of the AVG, we assumed that this would not have influenced the results. Also, the imaging protocol of the CTA scan was not optimized for segmentation of the vessel geometries. While the CTA dataset did allow for applying the proposed parametrization technique, image quality was insufficient for tracking the vessel wall at high detail over the complete length of the artery, graft and vein. Because irregularities in vessel wall and diameter were neglected and vessels were represented by constant diameter tubes in the proposed parametrization approach, the vessel walls and anastomotic geometry in the realistic AVG parametrizations can show some deviation from the actual in-vivo geometry. As a result, hemodynamics in the realistic AVG parametrizations used in this study may deviate slightly from reality. Though we believe that the impact of these assumptions is negligible compared to the errors made by using completely idealized AVG geometries, future studies should be performed to assess the effect of these assumptions on AVG hemodynamics.

This study demonstrates that AVG hemodynamics are considerably impacted by AVG parametrization. Because of the magnitude of the observed differences in hemodynamics between simulations with realistic or idealized AVG parametrizations, we hypothesize that, in general, hemodynamics in idealized AVG parametrizations do not accurately represent reality. However, since in this study AVG hemodynamics were only assessed in a realistic model of a single patient, albeit with the most often used configuration, more research is required to confirm or reject this hypothesis.

Our method for reconstructing realistic AVG geometries is based on centerline extraction. As such it is expected that this method can be applied to image data gathered with any 3D imaging technique as long as the general vessel path can be identified. As such, the proposed modelling approach likely provides flexibility in choosing the imaging technique that fits in best with the desired (clinical) workflow or study set-up. Nonetheless, the efficacy of the reconstruction algorithm on different input data should be investigated in future research.

In the current study all vessels were assumed to be rigid. In reality however, the vein and artery are often much more compliant than typically used PTFE grafts. The large mismatch between graft and vein compliance has been hypothesized to also play a role in AVG dysfunction [36–38]. In a recent study it was demonstrated that reducing the graft-vein compliance mismatch could indeed reduce the amount of transitional flow and detrimental WSS in the venous anastomosis of an AVG [39]. The reported effect of compliance mismatch in [39] is however often much smaller than the effect of changing AVG parametrization and boundary conditions reported in this study. As such, this suggests that for the purposes of this study the effect of wall compliance could indeed be neglected. Furthermore, the comparison with [39] demonstrates the importance of using modelling setups that are as realistic as possible, since the effect of modelling assumptions on simulation results might be larger than the difference that can be observed by comparing various graft concepts.

## 5 Conclusion

New dialysis graft designs are often hemodynamically evaluated using highly idealized geometries and boundary conditions. In this study we discuss two boundary condition models that allow for more realistic modelling of the peripheral vasculature. Furthermore we elaborate on methods to create realistic AVG parametrizations based on medical imaging data. We demonstrate that hemodynamics metrics related to graft failure are highly dependent on both the applied distal boundary conditions and the AVG geometry that is used. Consequently, the hemodynamics benefit of novel graft designs could be misrepresented when using idealized AVG geometries and/or non-physiological boundary conditions.

For future graft design optimization studies we therefore propose to use physiological boundary condition models and use realistic AVG geometries to demonstrate the efficacy of the graft.

## Supporting information

S1 Appendix (DOCX)
