# Supplementary material for: A realistic arteriovenous dialysis graft model for hemodynamic simulations
Source: PLoS One. 2022 Jul 21;17(7):e0269825. doi: 10.1371/journal.pone.0269825 (PMC9302782; doi:10.1371/journal.pone.0269825)
Supplement: S1 Appendix — (DOCX) [file pone.0269825.s001.docx]

1. Numerical arteriovenous coupling models implementation

This appendix details the implementation of the arteriovenous coupling models. First, the implementation of the arteriovenous model without collateral veins is described. Subsequently, the arteriovenous model is extended to include collateral veins. Both models were implemented using the methods described in Kroon et al. [22].

For the AVC-DB model, pressures and inward pointing flows were defined at the endpoints of each component of the arteriovenous model (Fig. A1). These pressures and flows are related as follows:

| $q_{1}=\frac{p_{1}-p_{2}}{Z{}_{\text{a}}}$ | $\begin{aligned} q_{6}=\frac{p_{4}-p_{3}}{Z{}_{\text{v}}}\#\left( 1 \right) \end{aligned}$ |
| --- | --- |
| $q_{2}=\frac{p_{2}-p_{1}}{Z{}_{\text{a}}}$ | $q_{7}=C_{\text{a}}\left( \frac{\partial p_{2}}{\partial t} - \frac{\partial p_{5}}{\partial t} \right)$ |
| $q_{3}=\frac{p_{2}-p_{3}}{R_{\text{p}}}$ | $q_{8}=C_{v}\left( \frac{\partial p_{3}}{\partial t} - \frac{\partial p_{6}}{\partial t} \right)$ |
| $q_{4}=\frac{p_{3}-p_{2}}{R_{\text{p}}}$ | $q_{9}=C_{\text{a}}\left( \frac{\partial p_{5}}{\partial t} - \frac{\partial p_{2}}{\partial t} \right)$ |
| $q_{5}=\frac{p_{3}-p_{4}}{Z{}_{\text{v}}}$ | $q_{10}=C_{\text{v}}\left( \frac{\partial p_{6}}{\partial t} - \frac{\partial p_{3}}{\partial t} \right).$ |

After taking into account that the sum of flow at each element junction equaled zero and after implementing all known pressures and flows, the following notation can be applied:

$$\begin{aligned} \bar{\boldsymbol{p}}=\left[ \begin{matrix} p_{1} \\ p_{2} \\ p_{3} \\ p_{4} \\ p_{5} \\ p_{6} \end{matrix} \right]=\left[ \begin{matrix} p_{1} \\ p_{2} \\ p_{3} \\ p_{4} \\ 0 \\ 0 \end{matrix} \right]\text{kPa},\quad\bar{\boldsymbol{q}}=\left[ \begin{matrix} q_{1} \\ q_{2}+q_{3}+q_{7} \\ q_{4}+q_{5}+q_{8} \\ q_{6} \\ q_{9} \\ q_{10} \end{matrix} \right]=\left[ \begin{matrix} Q_{\text{a}} \\ 0 \\ 0 \\ -Q_{\text{v}} \\ q_{9} \\ q_{10} \end{matrix} \right]\text{m}^{3}\text{s}^{-1}.\#\left( 2 \right) \end{aligned}$$

Using a second order backward differencing method, the relations in (1) can be rewritten as:

$\begin{aligned} \left[ \frac{3}{2\Delta t}\underline{\boldsymbol{C}}+\underline{\boldsymbol{R}} \right]{\bar{\boldsymbol{p}}}^{n+1}=\left[ \underline{\boldsymbol{C}}\left( \frac{2}{\Delta t}{\bar{\boldsymbol{p}}}^{n}-\frac{1}{2\Delta t}{\bar{\boldsymbol{p}}}^{n-1} \right) \right]+{\bar{\boldsymbol{q}}}^{n+1}. \#(3) \end{aligned}$ where matrices $\underline{\boldsymbol{C}}$ and $\underline{\boldsymbol{R}}$ are represented by:

$\begin{aligned} \underline{\boldsymbol{C}}=\left[ \begin{matrix} 0 & 0 & 0 & 0 & 0 & 0 \\ 0 & C_{\text{a}} & 0 & 0 & -C_{\text{a}} & 0 \\ 0 & 0 & C_{\text{v}} & 0 & 0 & -C_{\text{v}} \\ 0 & 0 & 0 & 0 & 0 & 0 \\ 0 & -C_{\text{a}} & 0 & 0 & C_{\text{a}} & 0 \\ 0 & 0 & -C_{\text{v}} & 0 & 0 & C_{\text{v}} \end{matrix} \right],\quad\underline{\boldsymbol{R}}=\left[ \begin{matrix} \frac{1}{Z_{\text{a}}} & -\frac{1}{Z_{\text{a}}} & 0 & 0 & 0 & 0 \\ -\frac{1}{Z_{\text{a}}} & \frac{1}{Z_{\text{a}}}+\frac{1}{R_{\text{p}}} & 0 & 0 & 0 & 0 \\ 0 & -\frac{1}{R_{\text{p}}} & \frac{1}{R_{\text{p}}}+\frac{1}{Z_{\text{v}}} & -\frac{1}{Z_{\text{v}}} & 0 & 0 \\ 0 & 0 & -\frac{1}{Z_{\text{v}}} & \frac{1}{Z_{\text{v}}} & 0 & 0 \\ 0 & 0 & 0 & 0 & 0 & 0 \\ 0 & 0 & 0 & 0 & 0 & 0 \end{matrix} \right],\#(4) \end{aligned}$and ${\bar{\boldsymbol{p}}}^{n+1}$, ${\bar{\boldsymbol{p}}}^{n}$, ${\bar{\boldsymbol{p}}}^{n-1}$ and ${\bar{\boldsymbol{q}}}^{n+1}$ represent the pressure and flow vectors at the next ($n+1$), current ($n$) and previous ($n-1$) timestep. It is assumed that $Q_{\text{a}}^{n+1}$ and $Q_{\text{v}}^{n+1}$ can be approximated by their current timestep value ($Q_{\text{a}}^{n}$ and $Q_{\text{v}}^{n}$). Linear system (7) is solved for each increment in time of the CFD simulation to obtain the pressures that are subsequently be prescribed as boundary condition on the arterial outlet and venous inlet of the CFD simulation.


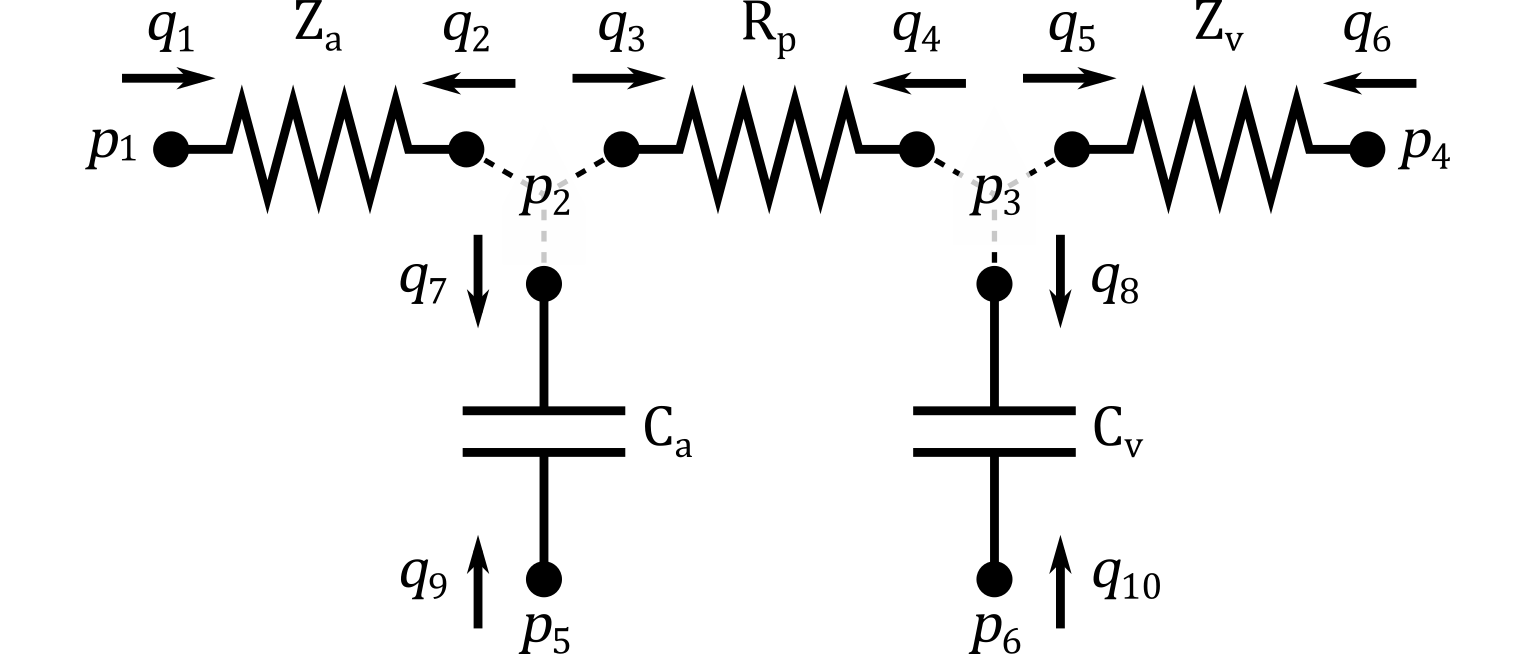


Figure A 1 Graphical representation of the numerical implementation of the AVC-DB model. At each nodal point, inward pointing flows are defined. Pressures are defined at each nodal junction and at the endpoints of the model. The arteriovenous model is connected to the arterial outlet of the 3D AVG model at the leftmost node, whereas the rightmost node is connected to the venous inlet of the 3D model.

The AVCE-DB model is implemented in a similar way as the AVC-DB model (Fig. A2) and yields two extra equations to be solved:

| $\begin{aligned} q_{11}=\frac{p_{4} - p_{7}}{R_{\text{c}}}\#\left( 5 \right) \end{aligned}$ |
| --- |
| $q_{12}=\frac{p_{7} - p_{4}}{R_{\text{c}}}.$  Furthermore: |

$$\begin{aligned} q_{6}+q_{11}=-\text{Q}_{\text{v}}.\#(6 \end{aligned})$$

Using the same temporal discretization as in (3), the expressions for $\bar{\boldsymbol{p}}$ and $\bar{\boldsymbol{q}}$ for the coupling model with collateral resistor are:

$\begin{aligned} \bar{\boldsymbol{p}}=\left[ \begin{matrix} p_{1} \\ p_{2} \\ p_{3} \\ p_{4} \\ p_{5} \\ p_{6} \\ p_{7} \end{matrix} \right]=\left[ \begin{matrix} p_{1} \\ p_{2} \\ p_{3} \\ p_{4} \\ 0 \\ 0 \\ 0 \end{matrix} \right]\text{kPa},\quad\bar{\boldsymbol{q}}=\left[ \begin{matrix} q_{1} \\ q_{2}+q_{3}+q_{7} \\ q_{4}+q_{5}+q_{8} \\ q_{6}+q_{11} \\ q_{9} \\ q_{10} \\ q_{12} \end{matrix} \right]=\left[ \begin{matrix} Q_{\text{a}} \\ 0 \\ 0 \\ -Q_{\text{v}} \\ q_{9} \\ q_{10} \\ q_{12} \end{matrix} \right]\text{m}^{3}\text{s}^{-1},\#\left( 7 \right) \end{aligned}$whereas the expressions for the matrices $\underline{\boldsymbol{C}}$ and $\underline{\boldsymbol{R}}$ are given by:

$$\begin{aligned} \underline{\boldsymbol{C}}=\left[ \begin{matrix} 0 & 0 & 0 & 0 & 0 & 0 & 0 \\ 0 & C_{\text{a}} & 0 & 0 & -C_{\text{a}} & 0 & 0 \\ 0 & 0 & C_{\text{v}} & 0 & 0 & -C_{\text{v}} & 0 \\ 0 & 0 & 0 & 0 & 0 & 0 & 0 \\ 0 & -C_{\text{a}} & 0 & 0 & C_{\text{a}} & 0 & 0 \\ 0 & 0 & -C_{\text{v}} & 0 & 0 & C_{\text{v}} & 0 \\ 0 & 0 & 0 & 0 & 0 & 0 & 0 \end{matrix} \right],\quad\underline{\boldsymbol{R}}=\left[ \begin{matrix} \frac{1}{Z_{\text{a}}} & -\frac{1}{Z_{\text{a}}} & 0 & 0 & 0 & 0 & 0 \\ -\frac{1}{Z_{\text{a}}} & \frac{1}{Z_{\text{a}}}+\frac{1}{R_{\text{p}}} & 0 & 0 & 0 & 0 & 0 \\ 0 & -\frac{1}{R_{\text{p}}} & \frac{1}{R_{\text{p}}}+\frac{1}{Z_{\text{v}}} & -\frac{1}{Z_{\text{v}}} & 0 & 0 & 0 \\ 0 & 0 & -\frac{1}{Z_{\text{v}}} & \frac{1}{Z_{\text{v}}}+\frac{1}{R_{\text{c}}} & 0 & 0 & -\frac{1}{R_{\text{c}}} \\ 0 & 0 & 0 & 0 & 0 & 0 & 0 \\ 0 & 0 & 0 & 0 & 0 & 0 & 0 \\ 0 & 0 & 0 & -\frac{1}{R_{\text{c}}} & 0 & 0 & \frac{1}{R_{\text{c}}} \end{matrix} \right].\#(8) \\ \end{aligned}$$

Similar to the AVC-DB model, (8) is solved for each time increment of the CFD simulation to calculate the pressures that are prescribed as boundary conditions on the arterial outlet and venous inlet of the CFD domain.


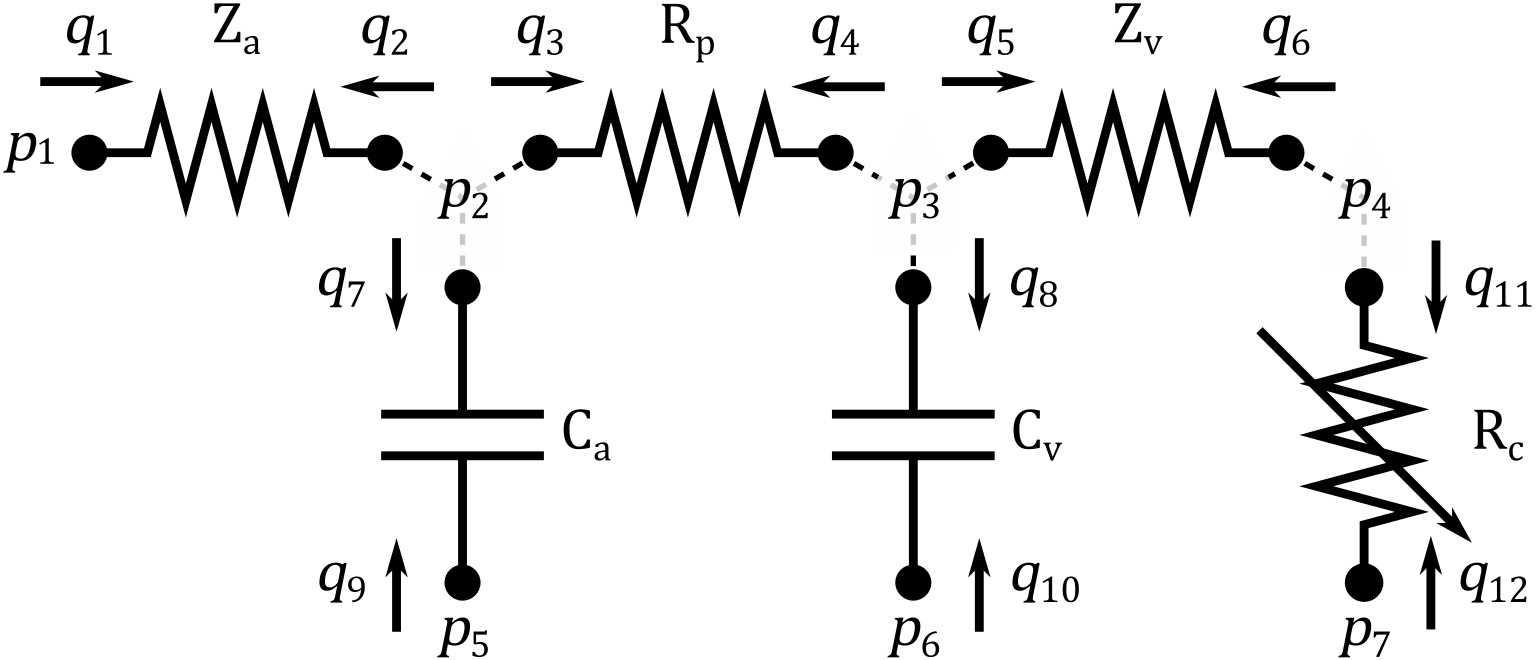


Figure A 2 Graphical representation of the numerical implementation of the AVCE-DB model. At each nodal point, inward pointing flows are defined. Pressures are defined each nodal junction and at the endpoints of the model. The arteriovenous model is connected to the arterial outlet of the 3D AVG model at the leftmost node, whereas the venous inlet of the 3D model is connected to the junction between $\text{Z}_{\text{v}}$ and $\text{R}_{\text{c}}$ **.**
